# Editorial: Neurological and Psychiatric Disorders in Endocrine Diseases

**DOI:** 10.3389/fendo.2015.00075

**Published:** 2015-05-12

**Authors:** Gianluca Tamagno, Jacques Epelbaum

**Affiliations:** ^1^Department of Endocrinology/Diabetes, Mater Misericordiae University Hospital, University College Dublin, Dublin, Ireland; ^2^UMR 894, Center for Psychiatry and Neuroscience, INSERM, Université Paris Descartes, Sorbonne Paris Cité, Paris, France

**Keywords:** eating disorders, exercise, gender identity, acromegaly, Cushing syndrome, polycystic ovary syndrome, obesity, hypogonadism, thyroid surgery, quality of life

In the last few decades, a number of reciprocal associations between endocrine system dysfunctions and neurological or psychiatric manifestations have appeared, and only in the minority of the cases this link has been fully elucidated (1). Unfortunately, the complexity, and sometimes also the severity, of the clinical issues and the limited availability of reliable experimental models for the study of the endocrine system–nervous system cross-interactions do not help the fast achievement of scientific progresses.

Isolated neurological or psychiatric symptoms as well as more complex disorders can occur in the setting of most endocrine diseases. Such manifestations can be the direct result of hyper- or hyposecretion of hormones from the endocrine glands or may occur as events secondary to the pathogenic mechanisms of the endocrinopathy, like in the case of autoimmunity affecting both endocrine glands and the brain (2). Moreover, the medical or surgical treatment of endocrine diseases can sometimes determine the occurrence of neurological or psychiatric syndromes. Some genetic aberrations can also lead to conditions affecting both the endocrine and the nervous system with a variety of possible manifestations. On the other side, many psychiatric or neurological diseases, including but not limiting to conditions, which affect the hypothalamus or the pituitary gland, may impair the physiological endocrine functions and demonstrate once again the intricate complexity of the psychoneuroendocrine connection.

Covering all basic, translational, and clinical aspects in a single Research Topic on the “Neurological and psychiatric disorders in endocrine diseases” cannot be realistically feasible. The scope is simply raising a number of interesting points from experts in the field to bring a contribution to the knowledge and maybe direct future research in this very intriguing area. Thus, we have collected reviews and original research articles, which may speak to a wide audience, including neurologists, psychiatrists, endocrinologists, and basic scientists in neuroscience, endocrinology, and metabolism.

In the clinical area, an interesting study assesses the plasma levels of the muscle-derived hormone irisin in anorexic women, without finding any significant correlation between irisin level and physical exercise (3). Another fascinating study analyzes the complexity of psychiatric and personality disorders in women with polycystic ovary syndrome, highlighting the issue of psychological distress in this patient group (4). A comprehensive picture of gender dysphoria, a psychiatric condition, which requires endocrine management, in Ireland is presented (5). In a short review, the most relevant reproductive, neurodevelopmental, and genetic aspects of hypogonadotropic hypogonadal syndromes are outlined (6). A German study indicates that psychopathology significantly predicts quality of life in patients with acromegaly and suggests that depressive symptoms and anxiety, being modifiable factors, may represent relevant targets for a broad treatment intervention in acromegalic patients (7). In a concise review, the psychiatric alterations, the neurocognitive impairment, and the altered quality of life affecting at some extent the majority of the patients with Cushing’s syndrome are summarized, and the authors highlight that resolution of hypercortisolism, a challenging and non-granted achievement, does not always lead to the complete remission of the neuropsychiatric changes or restore the quality of life (8). With an incursion in the surgical field, the complications potentially affecting the laryngeal nerves during thyroid surgery are extensively reviewed, suggesting that still the incidence of laryngeal nerves damage secondary to thyroid surgery cannot be suppressed but may be maintained in a low range with thorough surgical techniques and the use of intraoperative neuromonitoring (9). Three articles address the complex interactions between metabolism and neuropsychiatric symptoms. The first one focuses on biological differences between restrictive anorexia nervosa and constitutional thinness, a controversial concept to describe young girls who follow a normal diet and differ from restrictive anorexia nervosa on a number of endocrine parameters (10). At the opposite of the spectrum, the second one reviews the role of inflammatory processes in the neuropsychiatric comorbidity associated with obesity (11). Finally, the last one summarizes the fascinating link through ghrelin peptides between appetite/reward/growth hormone axis and psychiatric disorders (12).

## Conflict of Interest Statement

The authors declare that the research was conducted in the absence of any commercial or financial relationships that could be construed as a potential conflict of interest.
