# Corrigendum: “Editorial: Neurological and Psychiatric Disorders in Endocrine Diseases”

**DOI:** 10.3389/fendo.2015.00101

**Published:** 2015-06-17

**Authors:** Gianluca Tamagno, Jacques Epelbaum

**Affiliations:** ^1^Department of Endocrinology/Diabetes, Mater Misericordiae University Hospital, University College Dublin, Dublin, Ireland; ^2^UMR 894, Center for Psychiatry and Neuroscience, INSERM, Université Paris Descartes, Sorbonne Paris Cité, Paris, France

**Keywords:** eating disorders, exercise, gender identity, acromegaly, Cushing syndrome, polycystic ovary syndrome, obesity, hypogonadism, thyroid surgery, quality of life

We have added the following correction to the Editorial article, now including a reference to Kern et al. “Apo-ghrelin receptor (apo-GHSR1a) regulates dopamine signaling in the brain.”

Four articles address the complex interactions between metabolism and neuropsychiatric symptoms. The first one focuses on biological differences between restrictive anorexia nervosa and constitutional thinness, a controversial concept to describe young girls who follow a normal diet and differ from restrictive anorexia nervosa on a number of endocrine parameters (10). At the opposite of the spectrum, the second one reviews the role of inflammatory processes in the neuropsychiatric comorbidity associated with obesity (11). The third one summarizes the fascinating link through ghrelin peptides between appetite/reward/growth hormone axis and psychiatric disorders (12). Finally, the last one proposes a molecular mechanism through allosteric interactions between dopamine/DRD2 and GHSR1 receptors for controlling appetite and the uncontrollable hyperphagia associated with Prader–Willi syndrome (13).

Addition of reference:

13. Kern A, Grande C, Smith RG. Apo-ghrelin receptor (apo-GHSR1a) regulates dopamine signaling in the brain. *Front Endocrinol* (2014) 5:129. doi:10.3389/fendo.2014.00129

## Conflict of Interest Statement

The authors declare that the research was conducted in the absence of any commercial or financial relationships that could be construed as a potential conflict of interest.

