# Erratum: Inhibition of Gasdermin D-Mediated Pyroptosis Attenuates the Severity of Seizures and Astroglial Damage in Kainic Acid-Induced Epileptic Mice

**DOI:** 10.3389/fphar.2022.873984

**Published:** 2022-03-03

**Authors:** Frontiers Production Office

**Affiliations:** Frontiers Media SA, Lausanne, Switzerland

**Keywords:** epilepsy, GSDMD, pyroptosis, astrocyte, inflammation

Due to a production error, there was a mistake in Figure 1, Figure 2 and Figure 3 as published. The images for Figure 1, Figure 2 and Figure 3 were replaced with the images for **Supplementary Figure S1**, **Supplementary Figure S2**, and **Supplementary Figure S3**, respectively. The correct images for Figure 1, Figure 2 and Figure 3 appear below.

**FIGURE 1:**
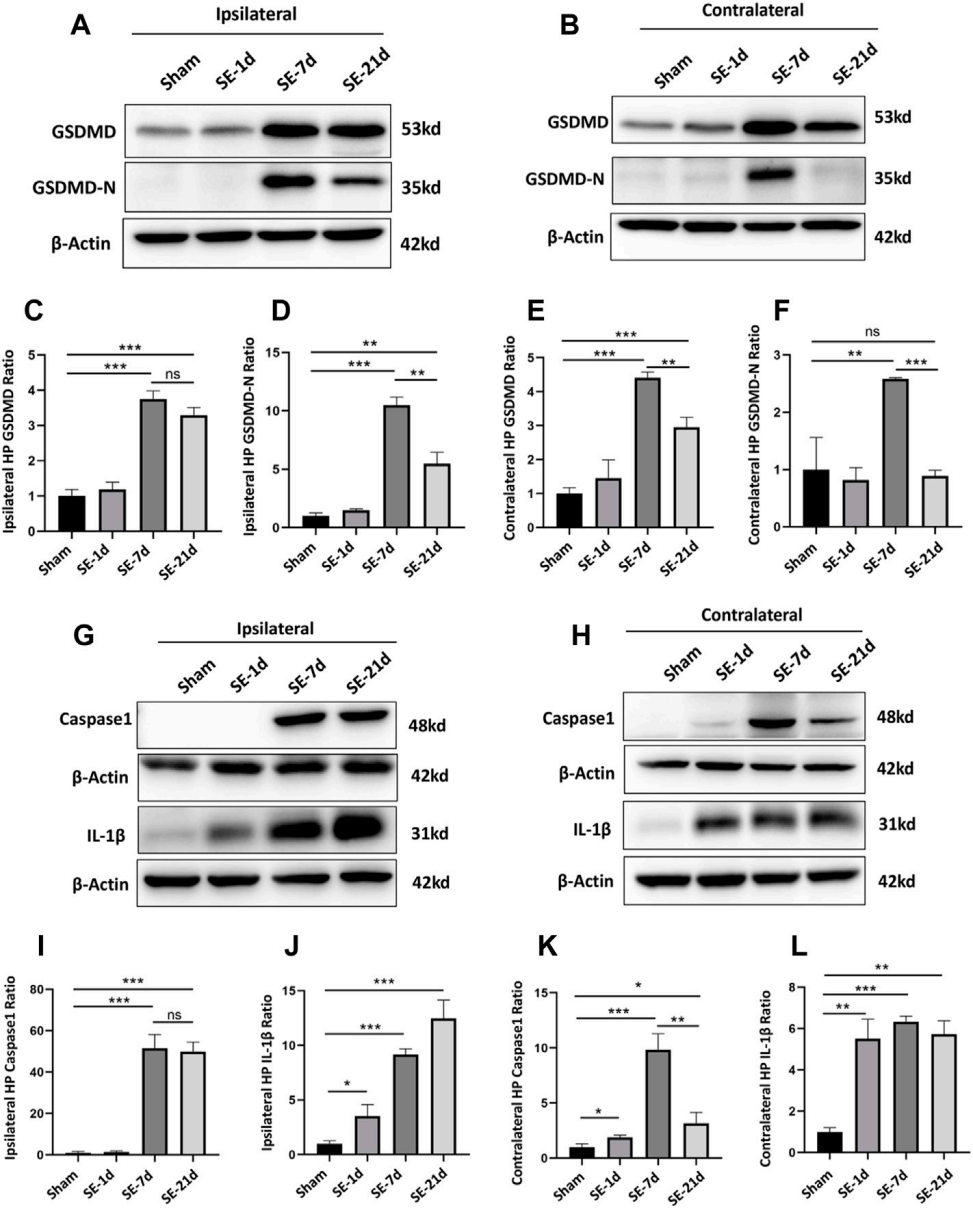
Expression of GSDMD and pyroptosis-related molecules was significantly increased after kainic acid-induced SE. **(A,B)** WB bands of GSDMD, GSDMD-N, and β-actin proteins in the ipsilateral and contralateral hippocampus. **(C–F)** Statistical analyses of GSDMD, GSDMD-N, and β-actin proteins in the ipsilateral and contralateral hippocampus. **(G,H)** WB bands of caspase-1, IL-1β, and β-actin proteins in the ipsilateral and contralateral hippocampus. **(I–L)** Statistical analyses of caspase-1, IL-1β, and β-actin proteins in the ipsilateral and contralateral hippocampus (*n* = 3 in each group, *p < 0.05, **p < 0.01, and ***p < 0.001).

**FIGURE 2:**
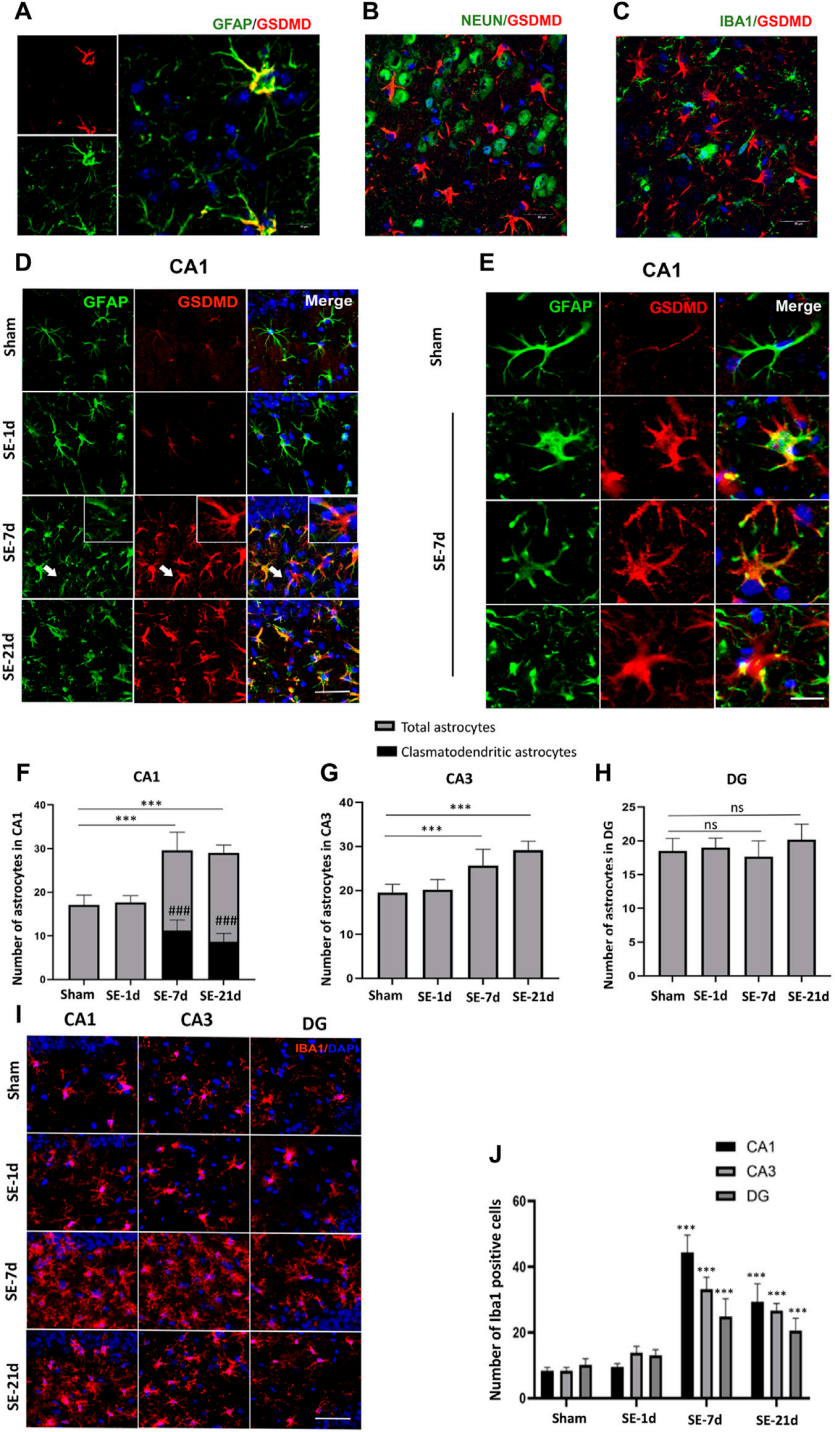
Clasmatodendritic astrocytes co-labeled with GSDMD after SE. **(A–C)** Representative images of GSDMD (red)/GFAP (green), GSDMD (red)/NeuN (green), and GSDMD (red)/Iba-1 (green) staining in hippocampal slices from mice at day 7 after kainic acid injection. **(D)** Microphotographs of GSDMD (red) and GFAP (green) staining in the CA1 region of the hippocampus in the sham, SE-1d, SE-7d, and SE-21d groups (bar = 50 µm). **(E)** Typical GSDMD-positive clasmatodendritic astrocytes in the CA1 region of the hippocampus at 7 days after SE (bar = 12.5 µm). **(F–H)** Statistical analyses of the number of GSDMD-positive clasmatodendritic astrocytes and total astrocytes in the CA1, CA3, and DG regions (*n* = 3 in each group, asterisks represent the total astrocytes in comparison with the sham group, *p < 0.05, **p < 0.01, and ***p < 0.001; well number represents the clasmatodendritic astrocytes in comparison with the sham group, ###*p* < 0.001). **(I)** Microphotographs of Iba1 (red) staining in the CA1, CA3, and DG regions of the hippocampus in the sham, SE-1d, SE-7d, and SE-21d groups. **(J)** Statistical analyses of the number of Iba1-positive cells in the CA1, CA3, and DG regions (*n* = 3 in each group, asterisks represent the comparison with the sham group, *p < 0.05, **p < 0.01, and ***p < 0.001).

**FIGURE 3:**
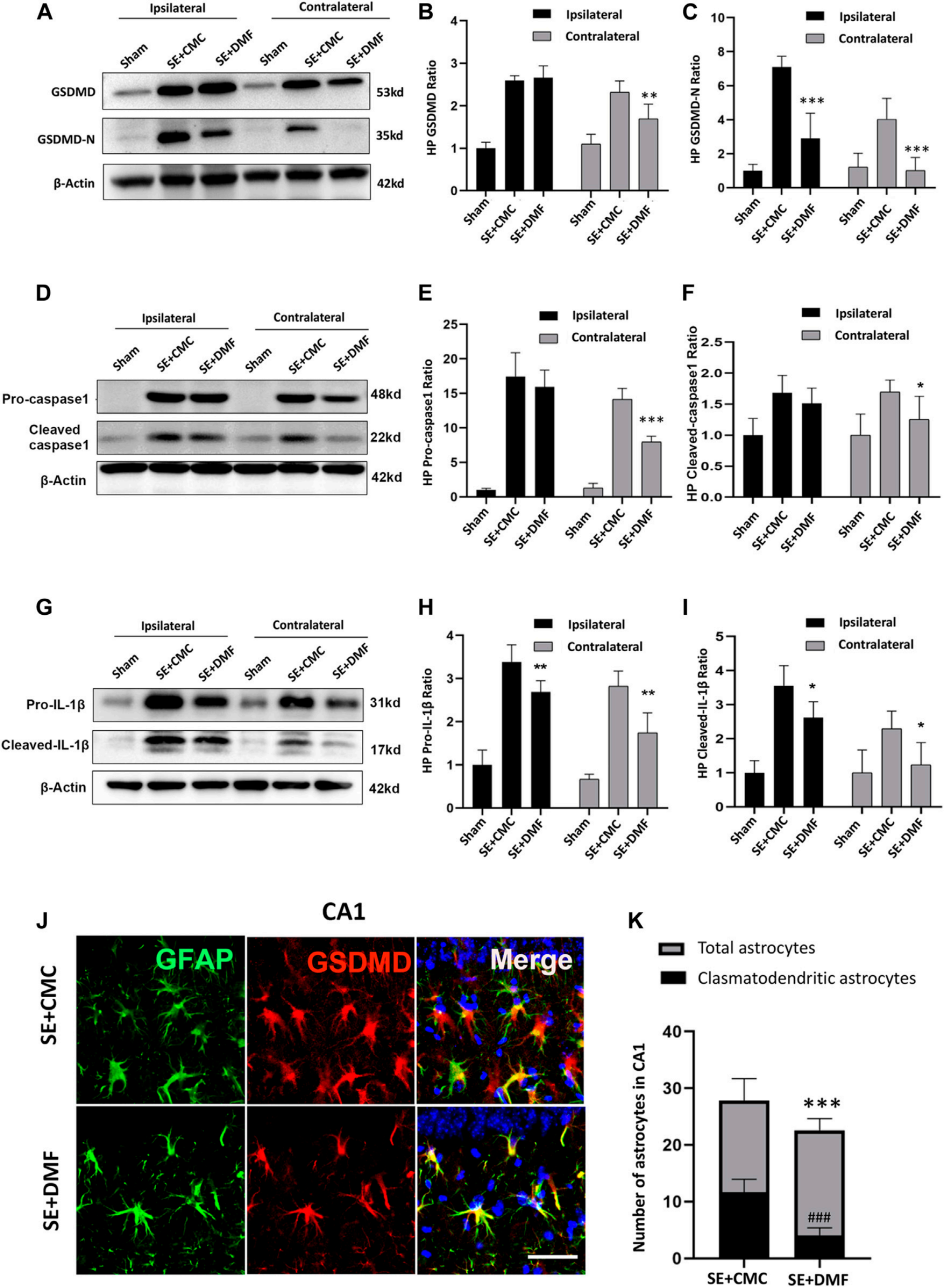
DMF intervention inhibited the expression of GSDMD-N and attenuated astrocytic clasmatodendrosis. **(A–C)** WB bands and statistical analyses of GSDMD, GSDMD-N, and β-actin proteins in the ipsilateral and contralateral hippocampus in the SE + CMC and SE + DMF groups (n = 6 in each group, asterisks represent the comparison with the SE + CMC group, *p < 0.05, **p < 0.01, and ***p < 0.001). **(D–F)** WB bands and statistical analyses of pro-caspase-1, cleaved-caspase1, and β-actin proteins in the ipsilateral and contralateral hippocampus (n = 6 in each group, asterisks indicate the comparison with the SE + CMC group, *p < 0.05, **p < 0.01, and ***p < 0.001). **(G–I)** WB bands and statistical analyses of pro-IL-1β, cleaved–IL-1β, and β-actin proteins in the ipsilateral and contralateral hippocampus (n = 6 in each group, asterisks indicate the comparison with the SE + CMC group, *p < 0.05, **p < 0.01, and ***p < 0.001). **(J)** Microphotographs of GSDMD (red) and GFAP (green) staining in the CA1 region of the hippocampus in the SE + CMC and SE + DMF groups. **(K)** Statistical analyses of the number of GSDMD-positive clasmatodendritic astrocytes and total astrocytes in the CA1 region in the SE + CMC and SE + DMF groups (n = 3 in each group, asterisks represents the total astrocytes in comparison with the sham group, ***p < 0.001; well number represents the clasmatodendritic astrocytes in comparison with the sham group, ###*p* < 0.001).

The publisher apologizes for this mistake. The original version of this article has been updated.

